# Photovoltaic Cell Surface Defect Detection Based on Wavelet-Aware Perception and Selective Reconstruction

**DOI:** 10.3390/mi17080907

**Published:** 2026-07-29

**Authors:** Li Yang, Simin Zhao, Hailong Duan, Xinrui Kan, Jing Yang, Jia Xing

**Affiliations:** 1 School of Automation and Electrical Engineering, Tianjin University of Technology and Education, Tianjin 300222, China; elsasundar@foxmail.com (S.Z.); nudt_dhl@sohu.com (H.D.); kanxinr@163.com (X.K.); 2 Tianjin Key Laboratory of Information Sensing and Intelligent Control, Tianjin 300222, China; 3 School of Information Technology Engineering, Tianjin University of Technology and Education, Tianjin 300222, China; abccode@163.com; 4 School of Science, Tianjin University of Technology and Education, Tianjin 300222, China; xingjiahappy@163.com

**Keywords:** photovoltaic cells, defect detection, wavelet transform, attention mechanism, selective reconstruction

## Abstract

Defect detection in electroluminescence (EL) images of solar cells remains challenging because defects are often small, exhibit subtle grayscale variations, and are obscured by complex cell-texture backgrounds. These conditions can attenuate defect features as they propagate through deep layers and make them difficult to distinguish from background patterns, thereby limiting the ability of detection models to accurately localize and classify defects. To address these issues, this study proposes a Wavelet-Aware Selective Reconstruction Network (WASR) for solar cell defect detection. A frequency-aware encoding strategy built around the Wavelet-Aware Downsampling Encoder (WADE) module preserves fine-grained defect cues by jointly modeling spatial and frequency-domain characteristics. In addition, a Cross-Scale Context Fusion Module (CCFM) improves multilevel feature transmission and fusion, whereas a Selective Reconstruction Detection Head (SRD-Head) performs selective feature reconstruction with residual enhancement. Together, these modules enhances defect perception capability under low-contrast conditions and complex backgrounds. Compared with the YOLO11 baseline, WASR increases mAP@0.5 by 2.4 percentage points, reduces the parameter count by 0.62 M, and improves inference speed by 23 FPS. The results show that WASR provides a favorable balance between detection accuracy and computational efficiency. Ablation experiments and visualization results further confirm the contribution of the proposed components under challenging conditions.

## 1. Introduction

Amid the global transition toward carbon neutrality, the photovoltaic (PV) industry has expanded rapidly as production scales and manufacturing efficiency improves [1,2]. As the basic power-generating elements of PV systems, solar-cell quality directly affects the energy conversion efficiency and long-term operational reliability of the entire system. Because fabrication involves several complex processes, including wafer preparation, dopant diffusion, and electrode sintering, surface defects can arise at multiple stages of production. These defects, such as small-scale cracks, finger interruptions, and black cores, degrade photoelectric conversion performance and may eventually cause substantial power loss or module failure. These consequences can become particularly severe during long-term operation. Reliable automated detection is therefore essential to improve PV product quality and maintain long-term system performance.

However, defects in photovoltaic electroluminescence (EL) images typically occupy small regions, exhibit low grayscale contrast, and are susceptible to background interference, making effective defect feature extraction challenging. Solar-cell defects were traditionally identified by manual visual inspection. However, this approach is limited by low efficiency and considerable subjectivity, making it unsuitable for the stringent quality requirements of large-scale industrial manufacturing. To automate inspection, several nondestructive techniques based on acoustic, laser, and optical sensing have been introduced [3,4,5,6]. Although these methods identify defects from physical signal responses, they generally require specialized equipment and substantial investment. Moreover, the stringent operating conditions often limit their applicability in complex manufacturing environments. Researchers then turned to classical image-processing methods, including threshold segmentation, edge detection, and texture analysis, often paired with machine-learning classifiers such as support vector machines [7,8]. Their performance, however, depends on handcrafted features with limited representation capacity, making discriminative feature extraction difficult when images contain low contrast, complex textures, or multiple defect types.

In recent years, deep learning methods have been widely applied to solar cell defect detection because they can learn task-relevant features automatically. Early work focused mainly on two-stage detection frameworks such as Faster R-CNN [9,10,11,12]. These frameworks improved accuracy through multiscale feature fusion. Their complex architectures and relatively slow inference, however, limit their use in real-time industrial inspection. As single-stage detectors have advanced, the YOLO series has been extensively adopted in industrial applications because of its high detection efficiency and real-time performance. To better preserve fine defect cues, several studies have redesigned feature-fusion structures and information-propagation paths for small-defect representation. For example, Xu et al. [13] introduced an SSD-based feature pyramid to improve feature fusion and multiclass defect detection. Meng et al. [14] optimized both the backbone network and feature fusion structure by leveraging the characteristics of EL images, allowing the model to maintain stable detection while operating in real time. Nevertheless, most of these methods still rely on convolutional neural networks for local feature modeling. In low-contrast, texture-rich scenes, background noise remains strongly correlated with defect regions, reducing the model’s ability to capture critical defect features. Attention mechanisms and global-context modeling have therefore been introduced to emphasize critical defect regions. Bao and Yuan [15] integrated an improved CBAM module into the YOLO framework, thereby enhancing the discriminative capability of both channel and spatial features. Subsequently, Mazen et al. [16] introduced the Global Attention Mechanism (GAM) into YOLOv5 and combined it with feature fusion strategies to improve multiscale defect detection performance. Acikgoz et al. [17] incorporated the Ghost module and a global attention mechanism into YOLOv7, significantly improving fine-crack recognition accuracy while maintaining computational efficiency. Despite these advances, subtle defect features can still deteriorate as they propagate through the network under challenging conditions, limiting detection reliability. As a mainstream real-time object detection framework, YOLO11 has been widely adopted in industrial defect inspection owing to its lightweight architecture and efficient inference capability. However, its performance remains limited in photovoltaic electroluminescence (EL) image analysis, particularly for small-scale defects such as micro-cracks and hidden cracks under complex background interference. The underlying challenge lies in the fact that photovoltaic defects usually contain abundant high-frequency information, including fine-grained edges, texture variations, and local structural patterns. Nevertheless, conventional deep neural networks may progressively weaken or discard these subtle features during hierarchical feature extraction and propagation, resulting in insufficient defect representation and reduced detection reliability.

To clarify why these limitations persist, researchers have examined deep neural networks through their feature-learning behavior. During training, deep neural networks generally learn low-frequency content before high-frequency details. This frequency bias weakens the representation of high-frequency cues associated with small defects [18]. This observation has prompted recent efforts to strengthen feature representation in the frequency domain. For example, Qin et al. [19] reformulated the channel attention mechanism using frequency-domain analysis and demonstrated that conventional global average pooling primarily captures low-frequency information. Their multispectral representation improved feature encoding and confirmed the value of frequency-domain information in visual tasks. In a related study, Wang et al. [20] constructed time–frequency representations of ultrasonic signals using variational mode decomposition and continuous wavelet transform techniques, and combined them with deep learning models to achieve high-precision defect recognition, demonstrating the effectiveness of frequency-domain features in defect representation. However, existing studies mainly focus on frequency-domain feature enhancement and time–frequency representation learning, while the role of frequency information in complex visual feature modeling remains under exploration.

**Figure 1:**
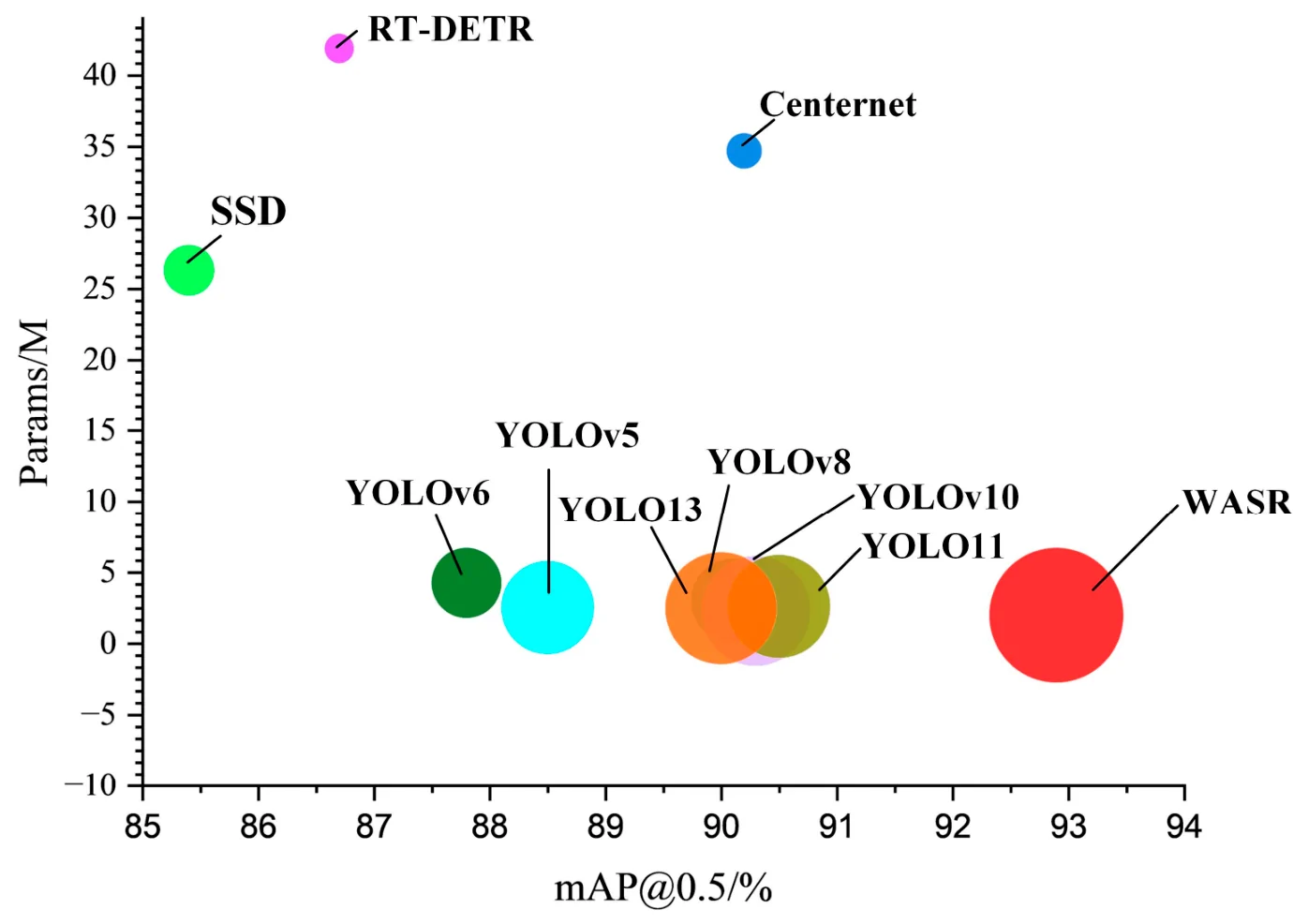
Comparison of different models.

To address small defects, low contrast, and complex textures in solar-cell EL images, this study presents a Wavelet-Aware Selective Reconstruction Network, termed WASR. The proposed method adopts YOLO11 as the baseline framework and introduces a task-oriented feature-processing strategy, where different components are collaboratively designed to address the limitations of defect detail preservation, background discrimination, and efficient feature fusion through optimized encoding, information fusion, and prediction processes. In practical photovoltaic manufacturing environments, detection accuracy is a critical factor for ensuring reliable defect identification, whereas inference speed determines whether the model can satisfy deployment requirements. Therefore, while maintaining high detection accuracy, a lightweight design is further introduced to reduce computational costs and achieve a better balance between detection performance and inference efficiency. The method was evaluated on the PVEL-AD photovoltaic-cell defect dataset released jointly by Hebei University of Technology and Beihang University. The results show that WASR provides a favorable balance among detection accuracy, model size, and inference speed. Figure 1 provides an intuitive comparison of model accuracy and efficiency. The x-axis reports mAP@0.5, the y-axis reports the parameter count (Params), and bubble size denotes inference speed (FPS). The main contributions of this work are as follows:

(1) A Wavelet-Aware Downsampling Encoder (WADE) is developed to preserve fine-grained defect cues that conventional downsampling may discard. By employing a parallel spatial–frequency architecture, the module jointly represents low-frequency structure and high-frequency detail, thereby strengthening small-defect characterization.
(2) A Cross-Scale Context Fusion Module (CCFM) reduces parameter and computational overhead while improving multilevel feature transmission and fusion. This design improves localization and stabilizes the detection of small defects.
(3) A Selective Reconstruction Detection Head (SRD-Head) improves classification and localization stability in low-contrast scenes with complex backgrounds. It selectively reconstructs channel and spatial features and uses residual enhancement to strengthen defect representations.

## 2. Methodology

**Figure 2:**
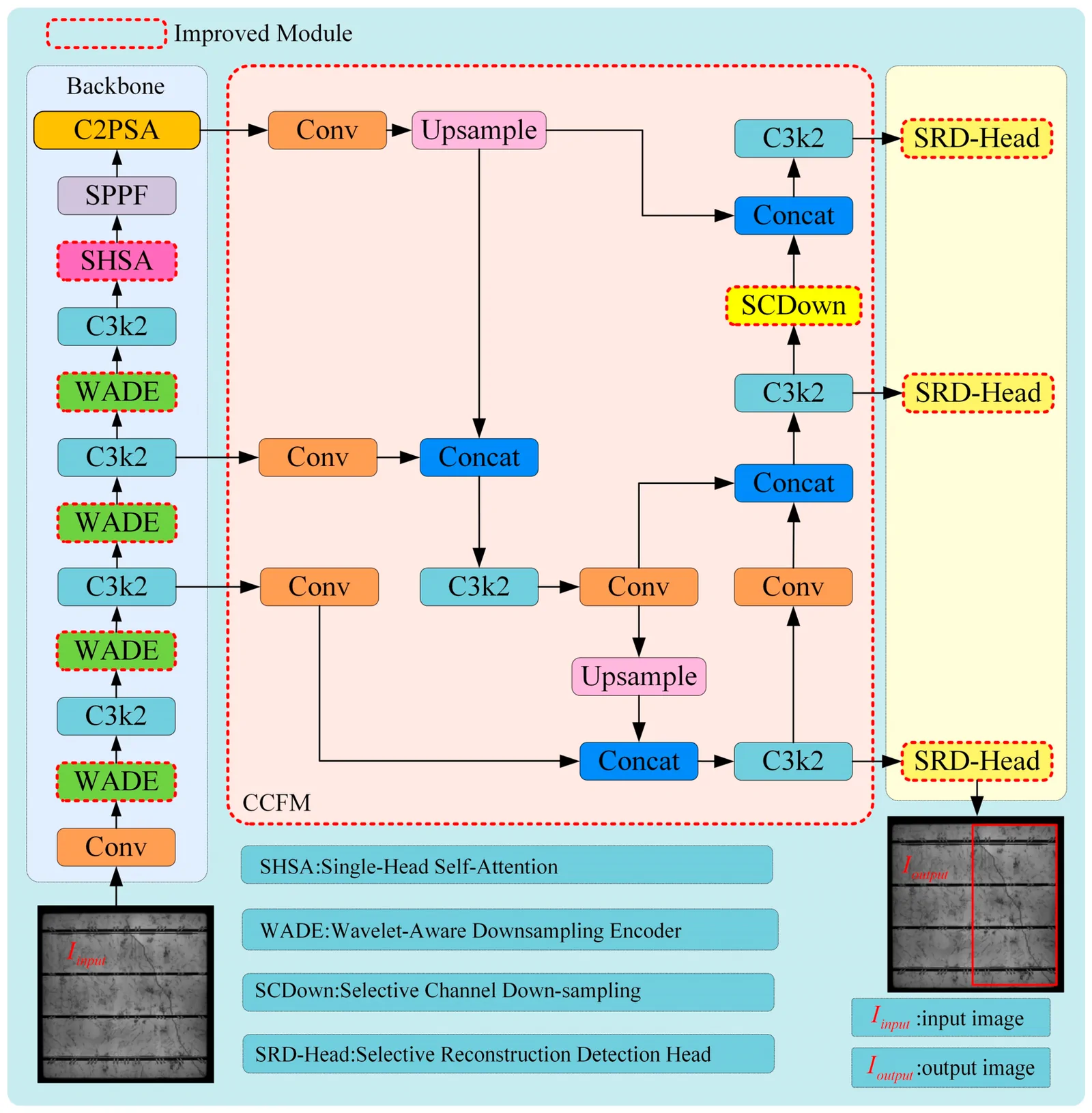
Overall architecture of WASR.

YOLO11 is an efficient object detector that balances detection accuracy and inference speed [21,22,23,24] across a range of detection tasks. Building on this lightweight baseline, this study develops a Wavelet-Aware Selective Reconstruction Network (WASR), whose overall architecture is illustrated in Figure 2. Rather than simply combining existing modules, WASR is designed according to the specific challenges of photovoltaic EL defect detection, including small defect sizes, subtle grayscale variations, and interference from complex cell-texture backgrounds. The proposed modules are collaboratively optimized at different stages of feature encoding, fusion, and prediction. At the encoding stage, the Wavelet-Aware Downsampling Encoder (WADE) is introduced to alleviate the loss of small and low-contrast defect information caused by conventional downsampling. By jointly modeling spatial and frequency-domain characteristics, WADE preserves high-frequency defect details and enhances weak defect representations. To suppress background interference, a lightweight Single-Head Self-Attention (SHSA) mechanism is embedded into the backbone. SHSA captures global contextual dependencies with limited computational overhead, improving defect–background discrimination. After initial feature enhancement, the Cross-Scale Context Fusion Module (CCFM) enhances hierarchical feature interaction while reducing redundant computation. Together with SCDown, CCFM improves multi-level feature transmission efficiency and maintains a favorable balance between detection performance and computational cost. After feature fusion, the Selective Reconstruction Detection Head (SRD-Head) further refines discriminative features through selective spatial and channel reconstruction, strengthening defect representations and suppressing irrelevant responses. Through this progressive design, WASR integrates task-oriented modules rather than simply stacking existing techniques. WADE preserves defect details, SHSA and SRD-Head enhance defect–background discrimination, and CCFM-SCDown enables efficient multi-scale feature fusion and lightweight deployment.

### 2.1. Wavelet-Aware Downsampling Encoder (WADE)

**Figure 3:**
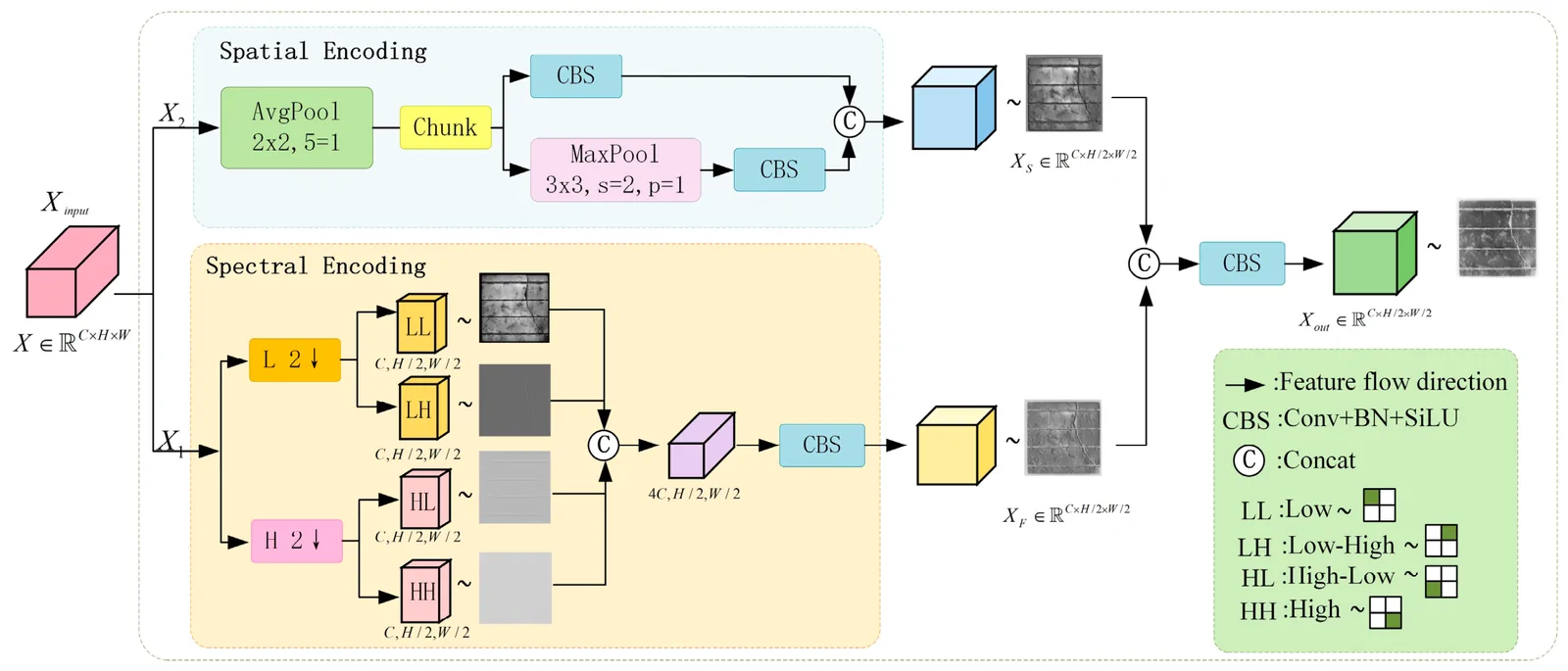
Architecture of the WADE module.

The Wavelet-Aware Downsampling Encoder (WADE) is designed to reduce high-frequency detail loss when small defects in photovoltaic EL images are downsampled (Figure 3). For defect detection, downsampling must reduce spatial resolution without discarding discriminative information. Conventional methods often remove fine details and consequently weaken feature separation between defects and background texture. WADE therefore uses a dual-branch encoder that integrates spatial and frequency-domain information. The combined representation preserves more informative cues throughout downsampling.

As shown in Figure 3, WADE processes an input feature map $X\in {\mathbb{R}}^{C\times H\times W}$ through two parallel branches: the spatial encoding branch and the frequency-domain encoding branch. The spatial branch applies adaptive downsampling. Average pooling first smooths the input features to reduce aliasing. The smoothed features are then divided into two channel groups. One path uses a stride-2 convolution to extract local structure, whereas the other applies max pooling to emphasize salient responses and a 1 × 1 convolution to normalize the channel dimension. Finally, the two sets of features are concatenated to produce a spatial representation $X_{S}$. This arrangement balances structural continuity with salient-region retention. In parallel, the frequency branch decomposes the input features using the discrete wavelet transform (DWT). The decomposition yields one low-frequency subband and three high-frequency subbands:

(1) {LL,LH,HL,HH}=DWT(X)

where X denotes the input feature map, DWT(⋅) denotes the discrete wavelet transform, LL denotes the low-frequency component containing the overall image structure, and LH, HL, and HH denote high-frequency components describing directional detail variations. The four subbands are then concatenated along the channel dimension to form the frequency-domain feature $X_{F}$. Unlike conventional downsampling, this operation retains high-frequency cues such as edges and textures that are important for fine-defect detection. The spatial- and frequency-domain outputs are subsequently concatenated and mapped by a convolution to obtain the final output:

(2) $$X_{out}=\phi (Concat(X_{S},X_{F}))$$

where ϕ(⋅) denotes a nonlinear mapping implemented by convolution, batch normalization, and activation. This fusion strategy combines complementary spatial and frequency information, producing more discriminative and robust feature representations.

### 2.2. Single-Head Self-Attention (SHSA)

**Figure 4:**
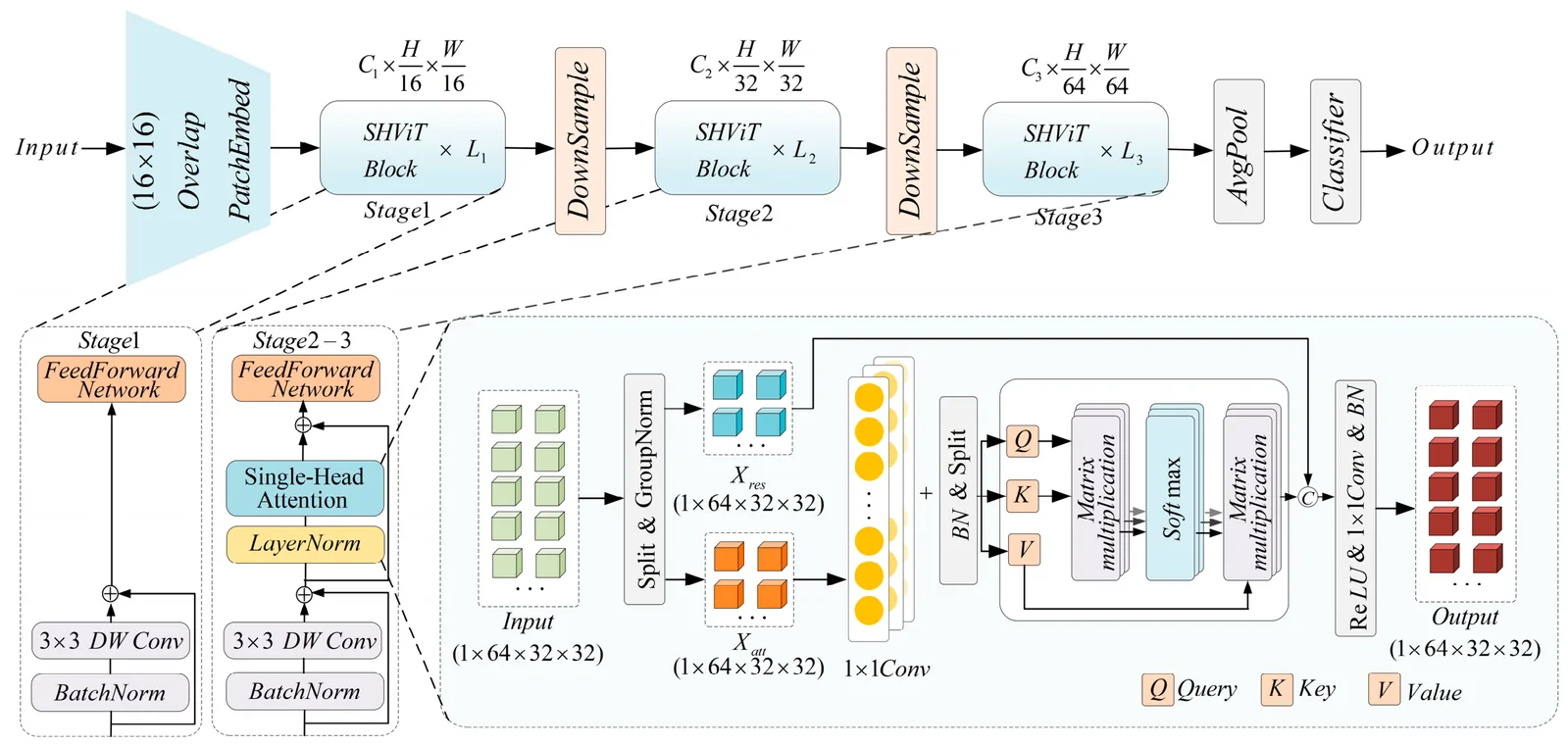
Structure of the SHSA module.

Convolutional neural networks (CNNs) extract local features effectively, but fixed receptive fields restrict long-range spatial modeling. In photovoltaic EL defect detection tasks, low-contrast defects often resemble surrounding textures and are difficult to separate using local cues alone. To introduce global context, the Single-Head Self-Attention (SHSA) module from the Single-Head Vision Transformer (SHViT) [25,26,27,28] is inserted into the deeper backbone layers (Figure 4).

The SHSA module takes a high-dimensional feature map $X\in {\mathbb{R}}^{C\times H\times W}$ as input, where H and W are the spatial dimensions and C is the number of channels. Split and GroupNorm first normalize the input and partition it along the channel dimension. The normalized tensor is then divided into two non-overlapping subsets, $X_{att}$ and $X_{res}$, according to a predefined channel ratio r. Here, $X_{att}\in {\mathbb{R}}^{H\times W\times C_{p}}$ denotes the subset of channels involved in the attention computation, whereas $X_{res}\in {\mathbb{R}}^{H\times W\times (C-C_{p})}$ forms a residual branch that retains the original local features. This partitioning forms a lightweight partial-channel attention path. The operation is given by Equation (3):

(3) $$X_{att},X_{res}=Split(X,[C_{p},C-C_{p}])$$

The residual bypass preserves critical local information without complicating channel partitioning and supports subsequent cross-scale fusion. For the attention branch $X_{att}$, the query (Q), key (K), and value (V) maps are obtained by linear projection. A scaled dot-product attention mechanism then models global interactions and produces the attention feature map ${\tilde{X}}_{att}$, which captures global spatial dependencies. This operation is expressed as follows:

(4) $${\tilde{X}}_{att}=Attention(X_{att}W^{Q},X_{att}W^{K},X_{att}W^{V})$$

where $W^{Q}$, $W^{K}$, and $W^{V}$ denote the corresponding learnable weight matrices, respectively. The feature map ${\tilde{X}}_{att}$ generated by global attention is concatenated with the residual channel subset $X_{res}$ along the channel dimension, to combine global dependencies with local details. A linear projection matrix $W^{O}$ then maps the fused feature back to the original channel dimension. The resulting output is defined as:

(5) $$SHSA(X)=Concat({\tilde{X}}_{att},X_{res})W^{O}$$

Compared with the conventional multi-head self-attention (MHSA) mechanism, SHSA adopts a single-head attention strategy with partial-channel computation, where global dependencies are modeled only on a subset of feature channels, while the remaining channels preserve local information through residual pathways. This design significantly reduces computational overhead and enhances the model’s capability to distinguish subtle defect features from background information. Because its input and output channel dimensions are identical, SHSA does not alter the original backbone propagation path. It can therefore be integrated into the backbone with minimal structural change to provide lightweight global-context enhancement.

### 2.3. Cross-Scale Context Fusion Neck

**Figure 5:**
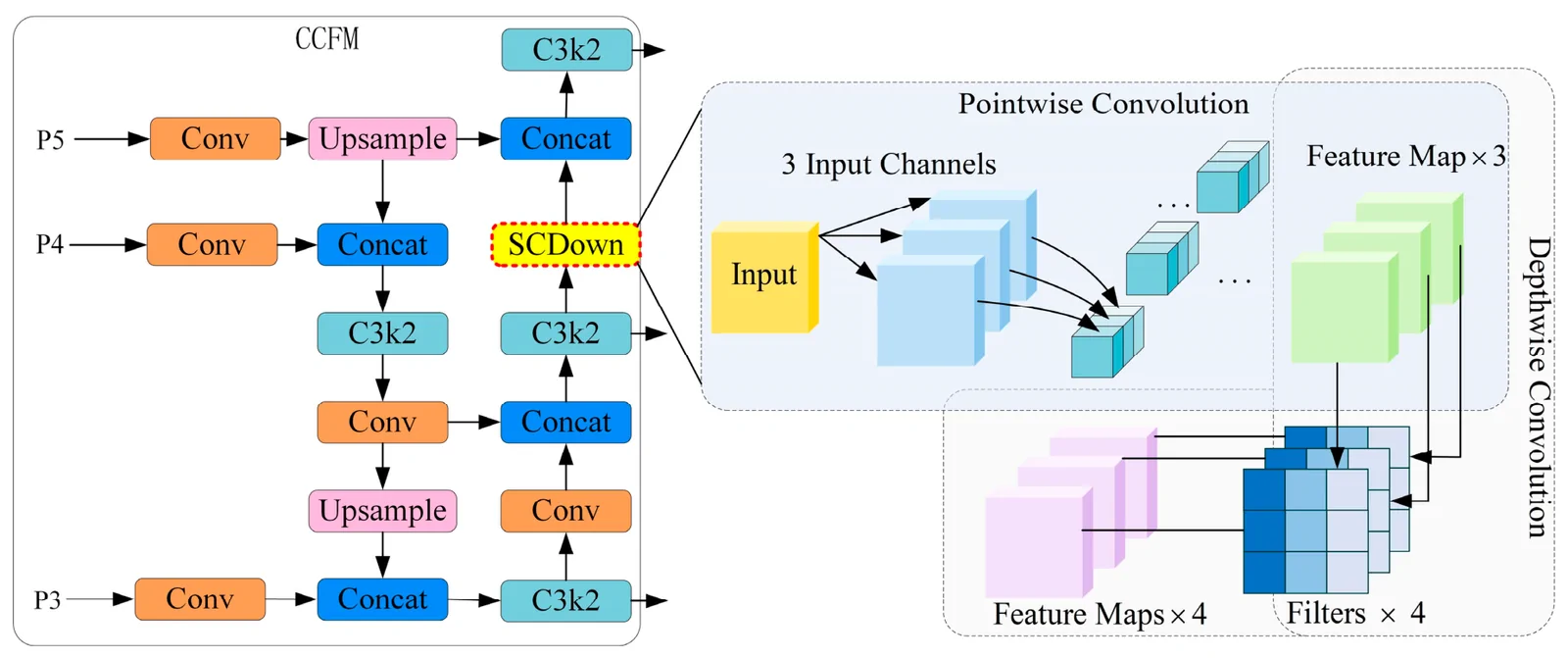
Architecture of CCFM.

In object detection tasks, the neck fuses multiscale features so that objects of different sizes can be represented effectively. Although SHSA improves global modeling, inefficient cross-level exchange and redundant computation still limit feature quality and overall efficiency. To address these limitations, a Cross-Scale Context Fusion Module (CCFM) is introduced, as shown in Figure 5. CCFM combines channel and spatial attention to emphasize informative responses selectively. Convolutions in the cross-scale branch align channels and remap features from different spatial scales. Before concatenation, convolutional layers all feature levels to 256 channels. This design reduces redundant computation, improves inference efficiency, and maintains dimensional consistency during cross-scale fusion. The embedded dual-attention mechanism also adjusts feature weights and suppresses background interference, improving cross-level interaction, scale adaptation, and small-defect detection.

Within the same neck, the SCDown module [29,30,31] replaces selected convolutional downsampling operations in the original PAN with lightweight units. Unlike a conventional stride-2 convolution, SCDown decouples channel and spatial processing. This reduces computational overhead while retaining discriminative information during downsampling. Combined with CCFM, SCDown enables more efficient fusion and more stable feature propagation.

### 2.4. Selective Reconstruction Detection Head (SRD-Head)

**Figure 6:**
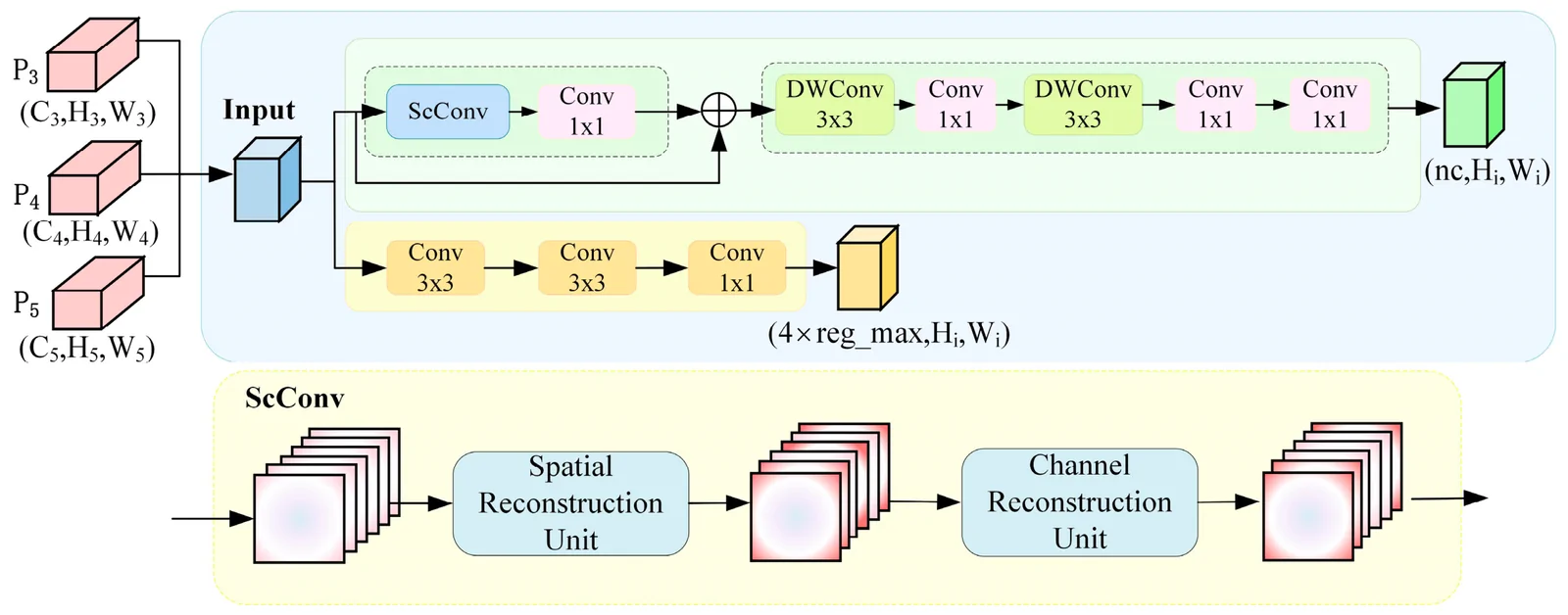
Structure of the SRD-Head.

Figure 6 shows the proposed Selective Reconstruction Detection Head (SRD-Head). The proposed SRD-Head uses ScConv for adaptive feature selection and reconstruction and adds residual enhancement [32,33,34,35]. The regression branch retains the conventional convolutional design. Multiple convolutional layers extract spatial cues and generate a bounding-box distribution of dimension 4×reg_max to model the discrete distribution of object boundaries. The classification branch follows a separate feature-enhancement path. The input feature map $X_{i}$ first enters an ScConv-based reconstruction module. This module connects a Spatial Reconstruction Unit (SRU) and a Channel Reconstruction Unit (CRU) in series. The SRU selectively reweights spatial responses by suppressing interference information from low-contribution spatial regions, thereby enhancing the feature representation of defect regions. The CRU enhances inter-channel information interaction and reconstruction to improve discriminative feature representation capability, thereby reducing the influence of background noise and contaminated textures on detection results. A residual connection then fuses the original and reconstructed features:

(6) $$X_{i}^{\prime }=X_{i}+F(\cdot )$$

In Equation (6), F(⋅) denotes the feature transformation implemented by ScConv and convolutional layers. The residual formulation strengthens the reconstructed response while preserving the original information, which stabilizes feature learning. The classification branch uses a lightweight depthwise-separable architecture in which two depthwise convolution (DWConv) layers are interleaved with pointwise convolutions to produce class predictions. The proposed SRD-Head improves feature discrimination while preserving a lightweight architecture.

## 3. Experimental Setup

### 3.1. Dataset

**Figure 7:**
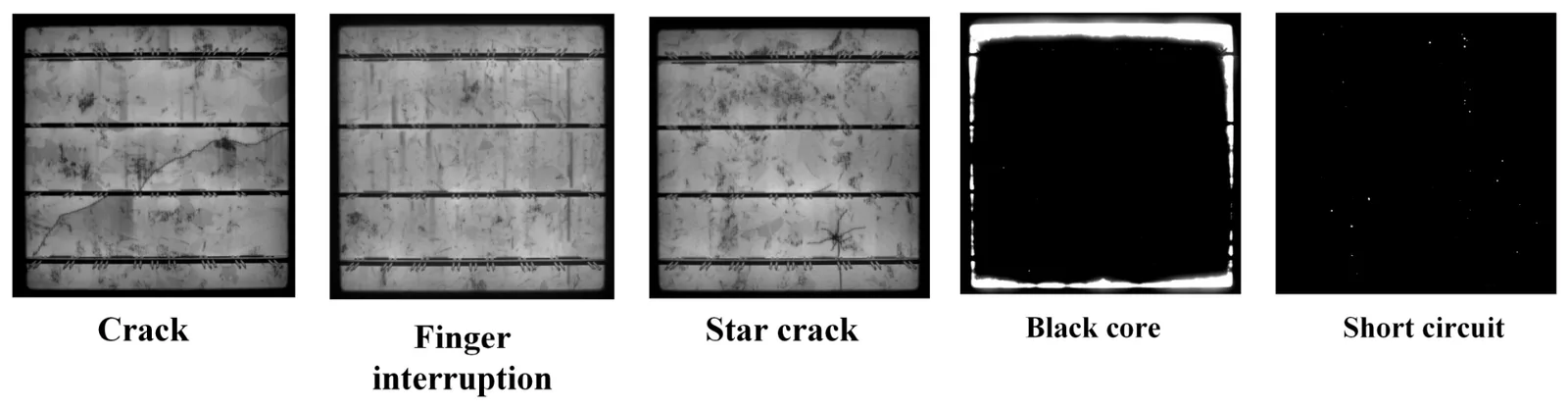
Sample images from the dataset.

Experiments used the PVEL-AD dataset [36,37,38,39], released jointly by Hebei University of Technology and Beihang University. The dataset contains annotated images acquired in industrial environments. After excluding categories with insufficient samples, a total of 6936 images were retained, including 4500 defect images and 2436 defect-free images. Based on defect morphology, the retained samples were grouped into five categories: Crack, Finger Interruption, Star Crack, Black Core, and Short Circuit. Example images from the dataset are presented in Figure 7.

**Figure 8:**
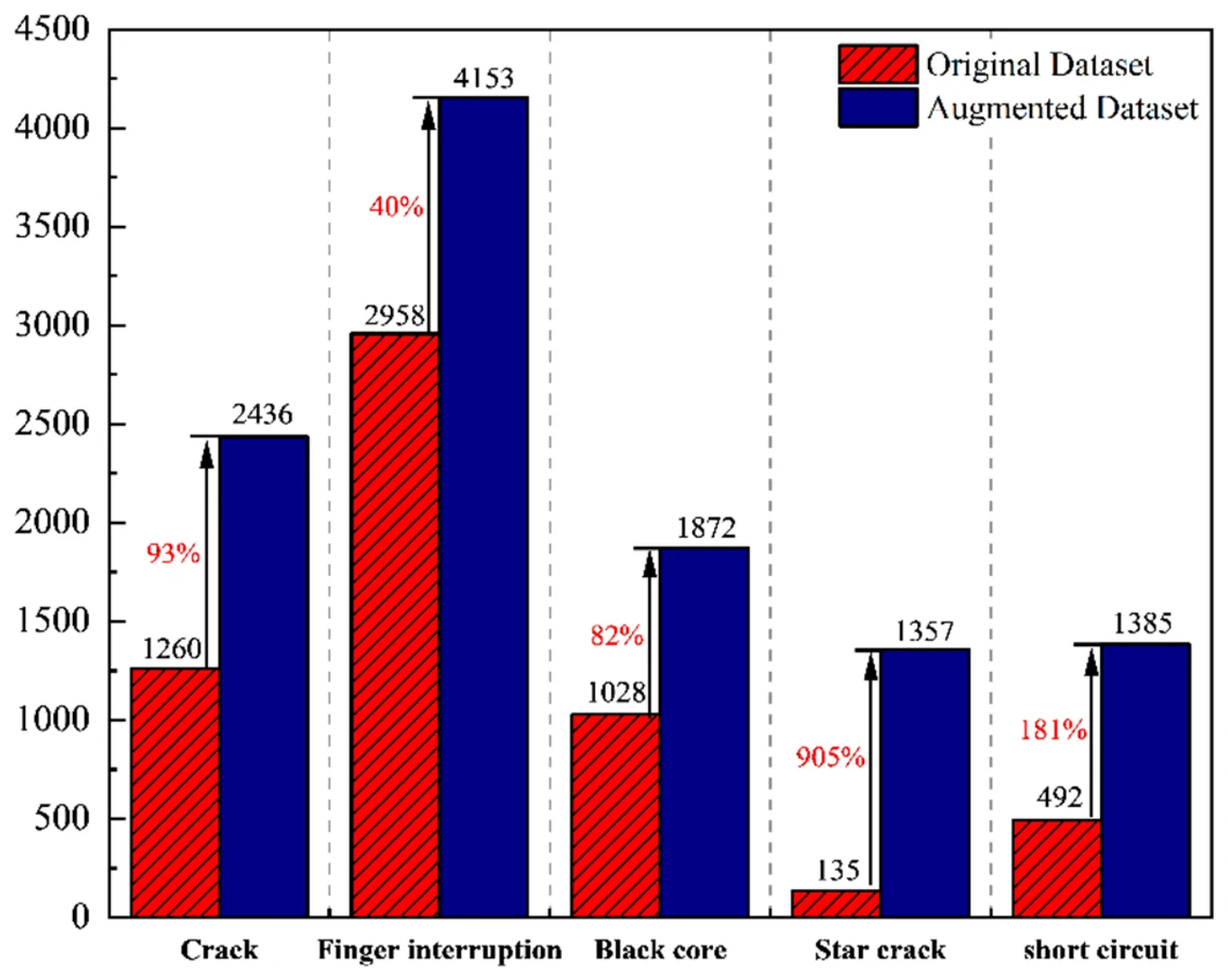
Class distributions before and after data augmentation.

Because the original dataset was relatively small, direct training could increase the risk of overfitting. The original samples also provided limited coverage of variations in defect scale, location, and illumination. A copy–paste strategy was therefore applied for data augmentation. The strategy increased the frequency and spatial diversity of defect instances, reduced class imbalance, and exposed the model to more varied background interference. The strategy increased the dataset from 6936 to 9636 images. The added samples broadened the data distribution while preserving image authenticity and annotation accuracy. Figure 8 compares the class distributions before and after augmentation. The increase was more pronounced for minority classes. The augmented dataset was divided into training, validation, and test sets at a ratio of 7:2:1, containing 6745, 1927, and 964 images, respectively. This partition provides sufficient training data and an independent test set for objective performance evaluation. This separation also improves the reliability and credibility of the reported experimental results.

### 3.2. Experimental Environment

All detection experiments were conducted under Windows 10 on a workstation with an Intel Core i9-12900K CPU (3.40 GHz), an NVIDIA GeForce RTX 3090 GPU with 24 GB of VRAM, and 128 GB of system memory. This configuration was used for both model training and evaluation. The software environment used Python 3.10 with PyTorch 1.13 as the deep-learning framework and CUDA 11.7 for GPU acceleration. Code development and debugging were performed in PyCharm 2020.3.3. Table 1 lists the training settings.

### 3.3. Evaluation Metrics

The model was evaluated in terms of detection accuracy and computational complexity. Precision (P), recall (R), and mAP@0.5 were used as the primary detection metrics. Precision is the proportion of positive predictions that are correct, whereas recall is the proportion of true targets that are detected. For each class, average precision (AP) is calculated as the area under the precision–recall (P–R) curve. Mean average precision (mAP) is the average AP over all classes. The metric mAP@0.5 uses an intersection-over-union (IoU) threshold of 0.5. Model compactness and real-time performance were further assessed using the number of parameters (Params), floating-point operations (FLOPs), and frames per second (FPS). Params measures model size, FLOPs estimates the cost of forward propagation, and FPS reports practical inference speed. The above multidimensional evaluation metrics are used to systematically analyze the model performance, and the corresponding formulas are as follows:

(7) $$P=\frac{TP}{TP+FP}$$

(8) $$R=\frac{TP}{TP+FN}$$

(9) $$AP=\int_{0}^{1}P(R)dR$$

(10) $$mAP=\frac{1}{c}\sum_{j=1}^{c}AP_{j}$$

(11) $$FPS=\frac{N}{T}$$

Here, TP is the number of true positives, FP is the number of false positives, and FN is the number of false negatives. C denotes the number of classes, N the number of processed images, and T the total inference time.

## 4. Results and Discussion

### 4.1. Detection Accuracy by Defect Category

**Figure 9:**
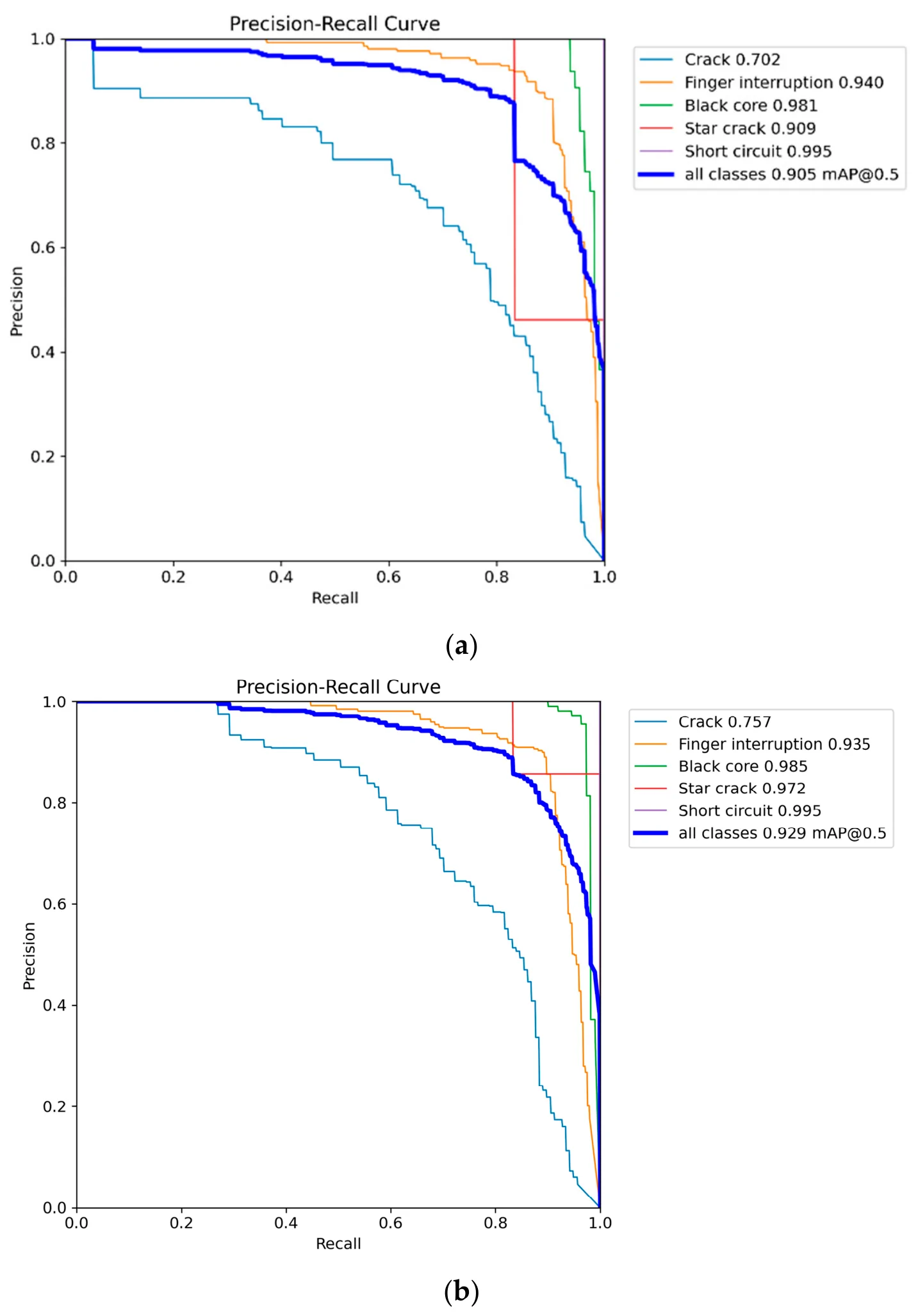
Precision–recall (P–R) curves for (**a**) YOLO11 and (**b**) WASR across the five defect categories. Panel (**a**) shows the classwise performance of YOLO11 for cracks, finger interruptions, black cores, star cracks, and short circuits, whereas panel (**b**) shows the corresponding WASR performance, including improved detection of challenging defects and more stable P–R curves.

Figure 9 compares the classwise P–R curves of the YOLO11 baseline and WASR. As shown by the overall curve distribution, the P–R curves of WASR are generally above those of YOLO11 for most categories, indicating improved overall detection performance. YOLO11 achieved an mAP@0.5 of 90.5%, whereas WASR reached 92.9%, an increase of 2.4 percentage points. The Crack category improved by 5.5 percentage points, and Star Crack showed the largest gain at 6.3 percentage points. Both defect types are generally small and contain edge-rich structures with subtle grayscale variation. WADE preserves their high-frequency details, while SHSA supplies global context in complex backgrounds. Together, the two modules improve fine-defect representation.

The 0.4-percentage-point gain for Black Core also suggests improved recognition of low-contrast defects. Finger Interruption showed a slight decline, although its overall accuracy remained high. These defects have relatively regular structures, and the baseline already recognized them well. Because WASR mainly targets complex backgrounds and weak fine-grained cues, its benefit for structurally regular defects is limited. Performance on Short Circuit remained unchanged. Overall, WASR maintains high accuracy across categories and provides the clearest gains for the more difficult defect classes, with smoother and more stable P–R curves.

### 4.2. Evaluation of the WADE Module

**Figure 10:**
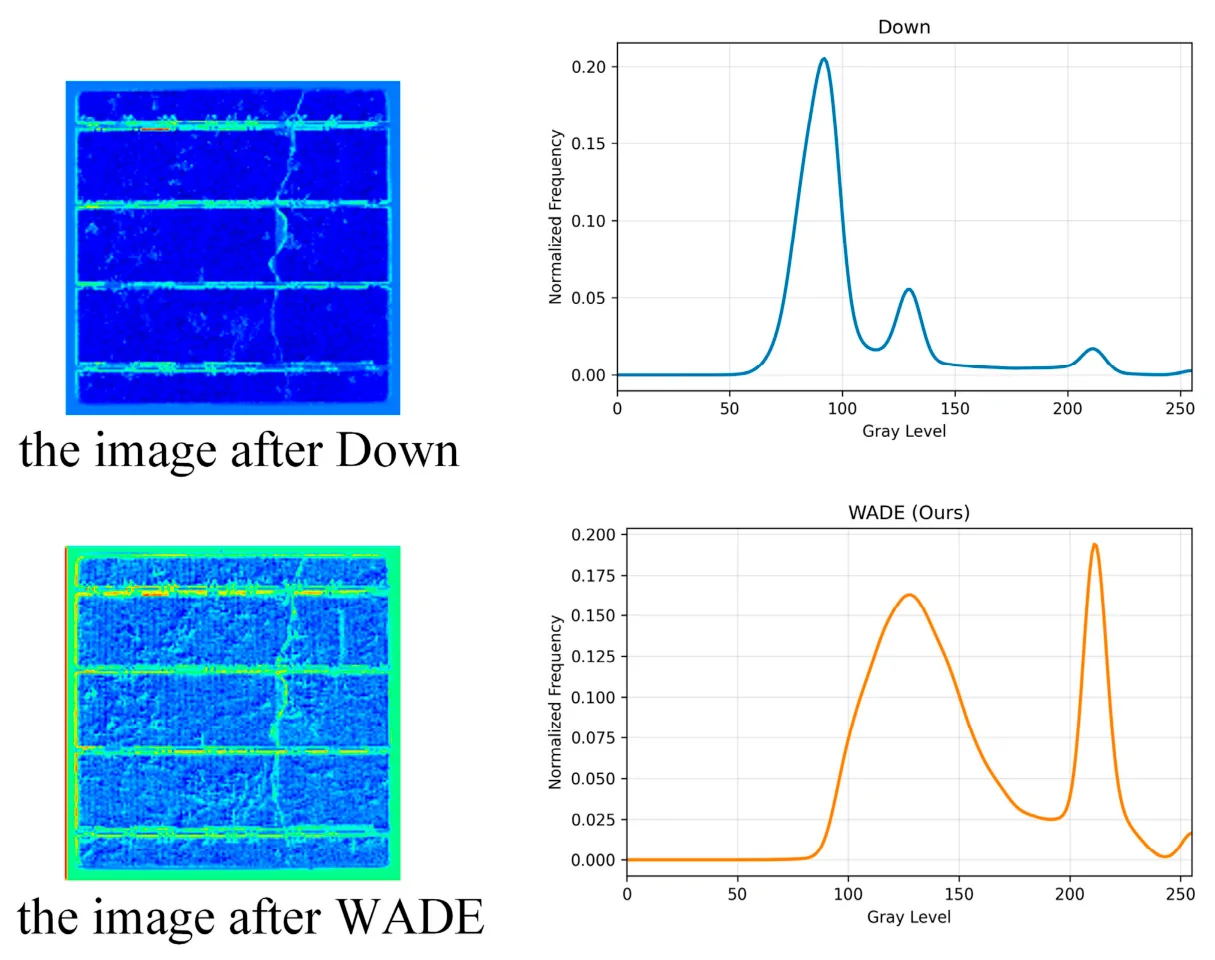
Grayscale distributions after conventional downsampling and WADE processing.

Figure 10 compares grayscale distributions after conventional downsampling and WADE processing. Conventional downsampling produced a largely unimodal distribution concentrated near the middle grayscale range, with limited separation between defects and background. WADE produced a clearer bimodal distribution: background pixels were concentrated at lower grayscale values, whereas defect pixels shifted toward higher values. The resulting separation increased the contrast between defect and background regions. These results indicate that WADE preserves defect information while improving feature contrast and separability for subsequent detection.

Table 2 compares conventional downsampling and WADE in terms of information content, contrast, and high-frequency detail preservation. WADE yielded substantially higher entropy, indicating that more feature information was retained. It also achieved a higher contrast value, reflecting stronger grayscale separation between defects and background. Its edge strength and edge ratio were 111.550 and 0.301, compared with 62.367 and 0.087 for conventional downsampling. The edge measurements confirm that WADE retains more fine-scale structure during downsampling. Overall, WADE preserves richer information, stronger contrast, and more high-frequency detail, providing a more discriminative input to subsequent detection layers.

### 4.3. Comparative Experiments

#### 4.3.1. Comparison with Advanced Algorithms

WASR was compared with several mainstream detectors on the same dataset. The results are summarized in Table 3. The methods differ in accuracy, complexity, and speed, illustrating the trade-off among these metrics.

Faster R-CNN used 41.5 M parameters and incurred high computational cost, yet achieved only 72.2% mAP@0.5. It therefore provides a poor accuracy–speed balance for this task. Single-stage detectors such as SSD and CenterNet provided a better efficiency–accuracy trade-off, but their overall performance remained limited. RT-DETR achieved relatively high recall but no clear advantage in overall accuracy or speed.

YOLO-series models, including YOLOv10, YOLOv11, and YOLOv13, provided a reasonable accuracy–efficiency balance but still struggled to separate subtle defects from background texture in low-contrast EL images. This ambiguity constrains further accuracy gains. WASR achieved the strongest overall results across the reported metrics. In particular, its mAP@0.5 reached 92.9%, significantly outperforming all comparison methods. With 1.96 M parameters, the smallest model in the comparison, WASR required 5.6 GFLOPs and reached 172 FPS. Thus, the gain in accuracy did not come at the expense of real-time performance. Overall, WASR provided the most favorable accuracy–efficiency balance. The combined modules therefore support accurate detection under complex industrial conditions. The coordinated interaction among the modules also improves detection stability.

**Figure 11:**
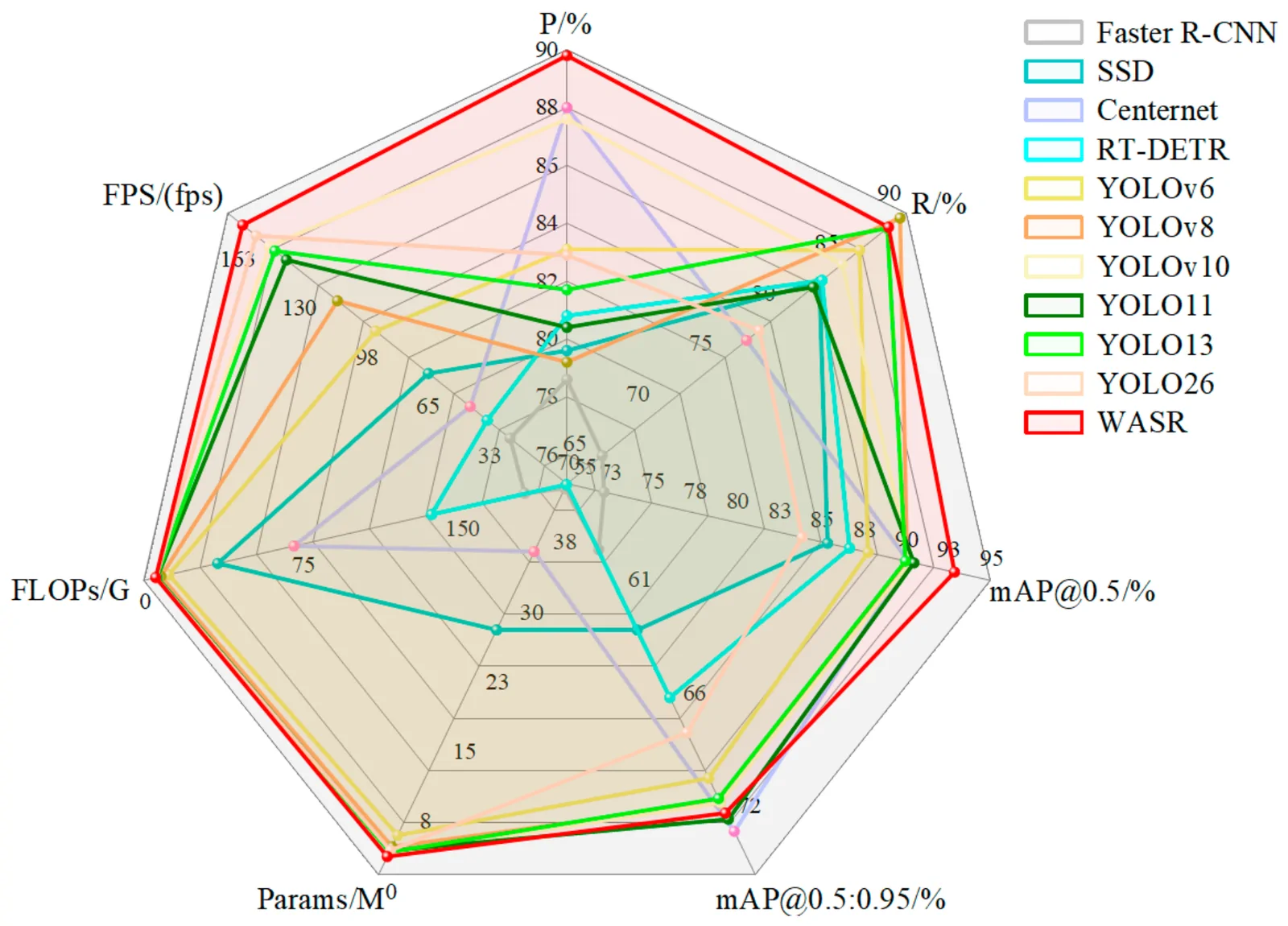
Overall performance comparison of the evaluated detectors.

Figure 11 presents a radar chart comparing the detectors in terms of accuracy, complexity, and inference speed.

#### 4.3.2. Comparison of Feature Fusion Methods

To verify the effectiveness of CCFM in multi-scale defect feature fusion, CCFM is compared with several typical feature fusion structures, including PAN-FPN, BiFPN, and Frefusion. The experimental results are presented in Table 4.

The data in Table 4 show that CCFM achieves an mAP@0.5 of 90.9%, which is slightly lower than Frefusion (91.3%). However, its parameter size is only 1.88 M, representing reductions of 27.1%, 33.8%, and 22.6% compared with PAN-FPN, BiFPN, and Frefusion, respectively. Meanwhile, the FLOPs are reduced to 5.3 G, and the inference speed reaches 175 FPS. Although Frefusion has a slight advantage in detection accuracy, it introduces relatively higher computational overhead. In contrast, CCFM achieves comparable detection performance with a smaller model size through lightweight feature interaction design, while improving inference efficiency. Therefore, considering detection accuracy, model complexity, and real-time deployment requirements, CCFM is more suitable for defect detection tasks in resource-constrained environments.

### 4.4. Ablation Study

Ablation experiments were conducted on the YOLO11 baseline to quantify the contribution of each module. Table 5 reports the results, with “√” denoting an incorporated module. Adding WADE in Experiment 1 increased mAP@0.5 to 92.8%. The parallel spatial and frequency branches allow WADE to retain local structure while incorporating frequency-domain cues. Their complementary outputs improve the separation of low-frequency structure and high-frequency detail. The gain was accompanied by a small increase in parameter count, reflecting the added encoding cost. In Experiment 2, SHSA improved fine-defect representation by adding global context. Experiment 3 reduced the parameter count to 1.88 M while largely preserving detection accuracy. This result indicates that the neck removes redundant computation while maintaining multilevel semantic interaction. In Experiment 4, SRD-Head increased mAP@0.5 to 91.9%. Its selective channel–spatial reconstruction and residual path improved classification and localization under complex backgrounds.

Additional combinations were evaluated to examine interactions among the modules. The results show that module gains are not additive and depend on how their feature-processing paths interact. WADE and SHSA each improved accuracy when used alone, but their combination in Experiment 5 reduced mAP@0.5 to 91.0%, suggesting redundant or competing feature responses. Experiments 6–9 removed individual modules from the complete architecture to estimate their contributions. Removing WADE or CCFM-SCDown produced the clearest performance declines, highlighting their roles in fine-detail extraction and multiscale propagation, respectively. Removing SHSA or SRD-Head also reduced performance, but to a lesser extent. The modules therefore address different stages of feature processing, although their interactions are not uniformly beneficial. It is worth noting that the primary contribution of CCFM-SCDown is not a direct increase in detection accuracy, but rather efficient feature fusion and computational reduction. As shown in Experiments 3 and 6–9, configurations containing CCFM-SCDown consistently achieved lower parameter counts than those without this module. For example, removing CCFM-SCDown from the complete model increased the parameter count from 1.96 M to 2.95 M (Experiment 9), accompanied by a decrease in detection performance. This result indicates that CCFM-SCDown effectively reduces redundant feature processing and improves multiscale feature propagation with lower computational overhead. Therefore, its contribution is reflected not only in accuracy improvement but also in achieving a better balance between detection performance and model complexity. The complete model in Experiment 10 achieved 92.9% mAP@0.5 with 1.96 M parameters. This configuration provides the best overall balance between accuracy and model size for practical deployment.

### 4.5. Generalization and Robustness Analysis

#### 4.5.1. Cross-Dataset Validation

**Figure 12:**
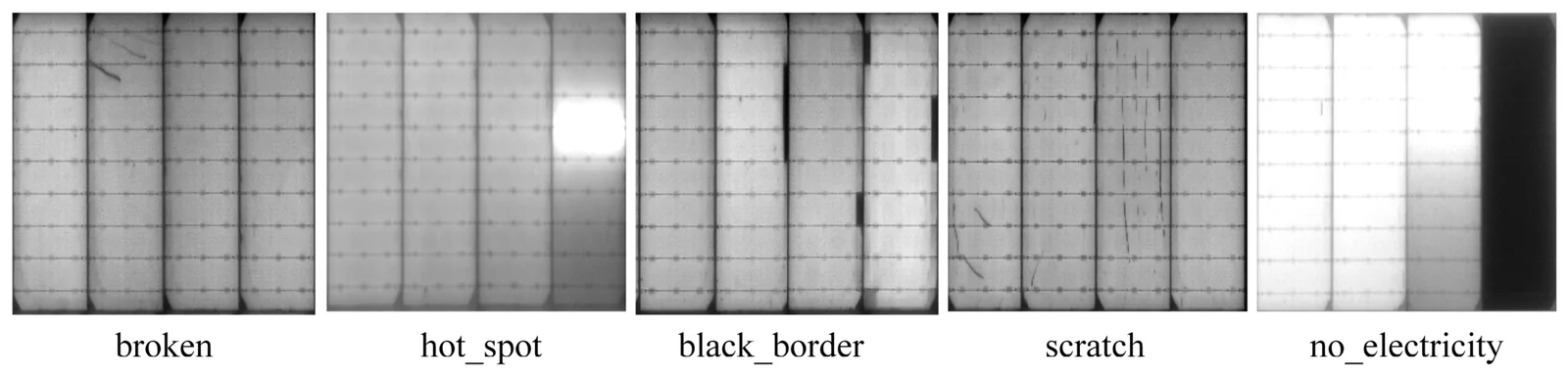
Representative images from the PV-Multi-Defect dataset.

To examine performance under a different data distribution, WASR was evaluated on PV-Multi-Defect, an infrared thermography dataset of monocrystalline silicon modules. Released by a research team at Central China Normal University, the dataset contains 1106 infrared thermographic images uniformly cropped to a resolution of 600 × 600 pixels. It covers five defect categories commonly observed during power-station inspection: hot spots, scratches, open circuits, dark regions, and damage defects, as illustrated in Figure 12. All models used the same training hyperparameters and inference settings. WASR was compared with YOLOv5, YOLOv6, YOLOv10, YOLO11, YOLO26, RT-DETR, and Faster R-CNN. Precision, recall, mAP@0.5, and mAP@0.5:0.95 were used for quantitative evaluation. Table 6 reports the complete results.

WASR obtained the highest PV-Multi-Defect scores, with precision, recall, mAP@0.5, and mAP@0.5:0.95 values of 82.6%, 75.0%, 83.4%, and 56.9%, respectively (Table 6). Compared with YOLO11, the corresponding gains were 3.3, 1.6, 2.5, and 2.2 percentage points. These results indicate that WASR remains effective under the tested alternative data distribution and has promising cross-dataset applicability. The gains therefore support favorable cross-dataset generalization under the tested conditions.

#### 4.5.2. Random-Seed Experiments

Repeated training with different random seeds was used to assess the stability of the observed gains. YOLO11 and WASR were each trained with three seeds while the data split, training strategy, and hyperparameters were held constant. Table 7 reports the mean ± standard deviation across the three runs.

Across the three random seeds, WASR outperformed YOLO11 on every reported metric (Table 6). Its standard deviations for mAP@0.5 and mAP@0.5:0.95 were 0.3 and 0.7, below the YOLO11 values of 1.1 and 0.84. The mean mAP@0.5 of 91.6% was only 1.3 percentage points below the fixed-seed result in Table 3, whereas the corresponding YOLO11 result decreased by 1.9 percentage points compared with its fixed-seed performance, indicating limited sensitivity to random initialization. Thus, the improvement was consistent across the tested seeds. The low run-to-run variability further supports the stability and reliability of the observed performance improvement.

#### 4.5.3. Failure Case Analysis

**Figure 13:**
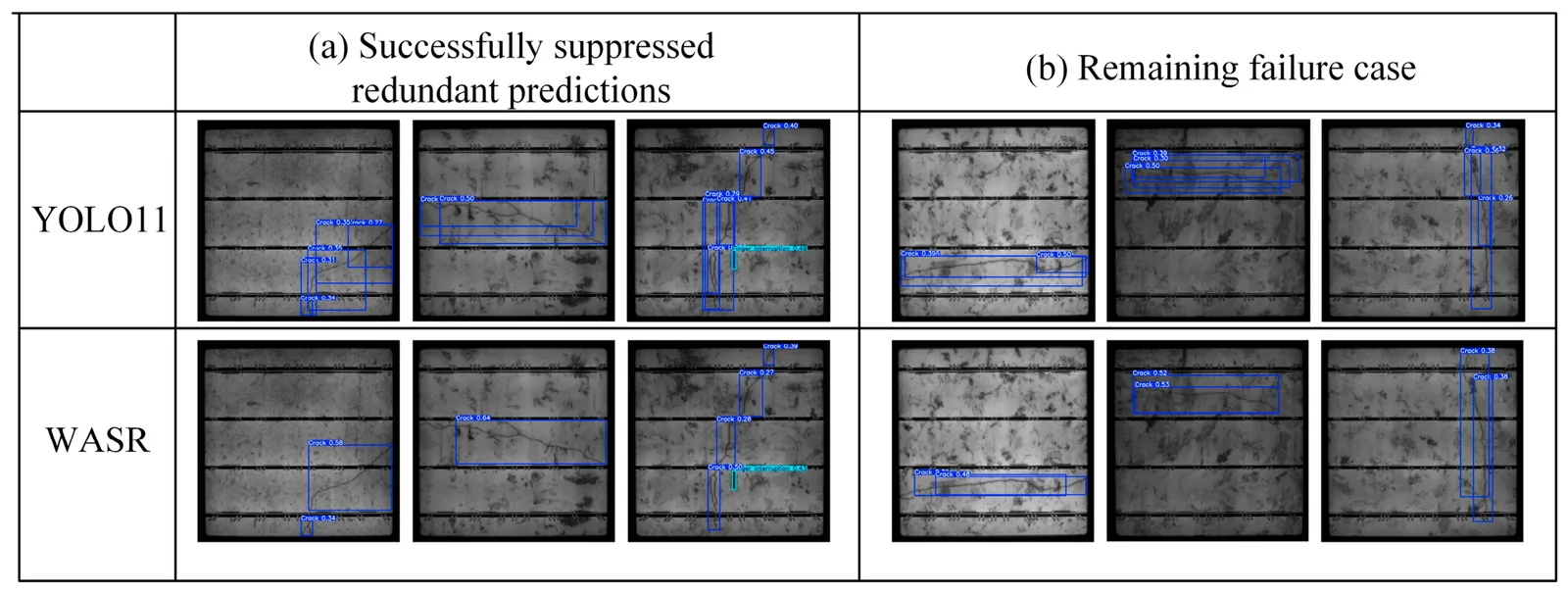
Representative failure case for an elongated crack.

Despite improved fine-detail representation and multiscale fusion, WASR still produces localization errors for a few exceptionally long, continuous cracks (Figure 13). These cracks are slender, continuous, and spatially extensive. Similar responses along different crack segments can generate several nearby predictions for one target. Their large-aspect-ratio boxes can also have low pairwise intersection over union (IoU), even when they refer to the same crack. IoU-based non-maximum suppression (NMS) may therefore fail to merge all redundant predictions for these slender targets. Nevertheless, WASR produces fewer redundant responses than the YOLO11 baseline. For the sample in Figure 13b, YOLO11 generates more repeated boxes, whereas WASR retains only a few, indicating improved but still incomplete localization consistency. The case highlights a remaining limitation of bounding-box-based detection for elongated and irregular targets. Future work may incorporate shape-aware constraints, center-distance criteria, or slender-target post-processing to improve crack localization and suppress redundant boxes.

### 4.6. Performance Evaluation and Validation of the Small-Scale Object Detection Model

Detection precision across object scales was evaluated using two partitioning schemes: fixed area thresholds and area-distribution percentiles. Table 8 and Table 9 report precision under the two schemes.

Under the fixed-threshold scheme, objects smaller than 1089 pixels (0.03 × 640 × 640) were classified as small, objects between 1089 and 3276 pixels (0.03–0.12) as medium, and larger objects as large. Under this scheme, WASR improved precision at all three scales. Although the improvement was more significant for medium-sized objects, detecting small objects remains more challenging due to their limited size, weak features, and susceptibility to downsampling. Therefore, the improvement achieved on small objects, with precision increasing from 78.3% to 84.9%, is particularly meaningful. This improvement is consistent with WADE preserving high-frequency detail through joint spatial–frequency encoding.

Because defect sizes are unevenly distributed, a second analysis grouped targets by area percentiles. The top 40% of targets by area were labeled small, the 40–80% range medium, and the remainder large. The 40th- and 80th-percentile thresholds were 1089.06 and 304,216.41 pixels, respectively. This scheme reflects the empirical area distribution rather than fixed thresholds. Under this criterion, WASR increased small-object precision from 83.4% to 86.7%. Gains at all scales show that the result is not limited to a single partitioning rule. Notably, the fixed small-object threshold of 0.03 × 640 × 640 is approximately 1089 pixels, nearly identical to the 40th-percentile value of 1089.06 pixels. The fixed threshold therefore aligns reasonably well with the observed target-size distribution. Because one scheme uses absolute area and the other uses percentiles, their group assignments still differ and produce different precision values.

Overall, WASR improves small-object precision under both scale-partitioning strategies, supporting its use for fine photovoltaic EL defects. Its consistent advantage across both partitioning schemes further confirms the method’s effectiveness and practical applicability in complex photovoltaic EL defect detection.

**Figure 14:**
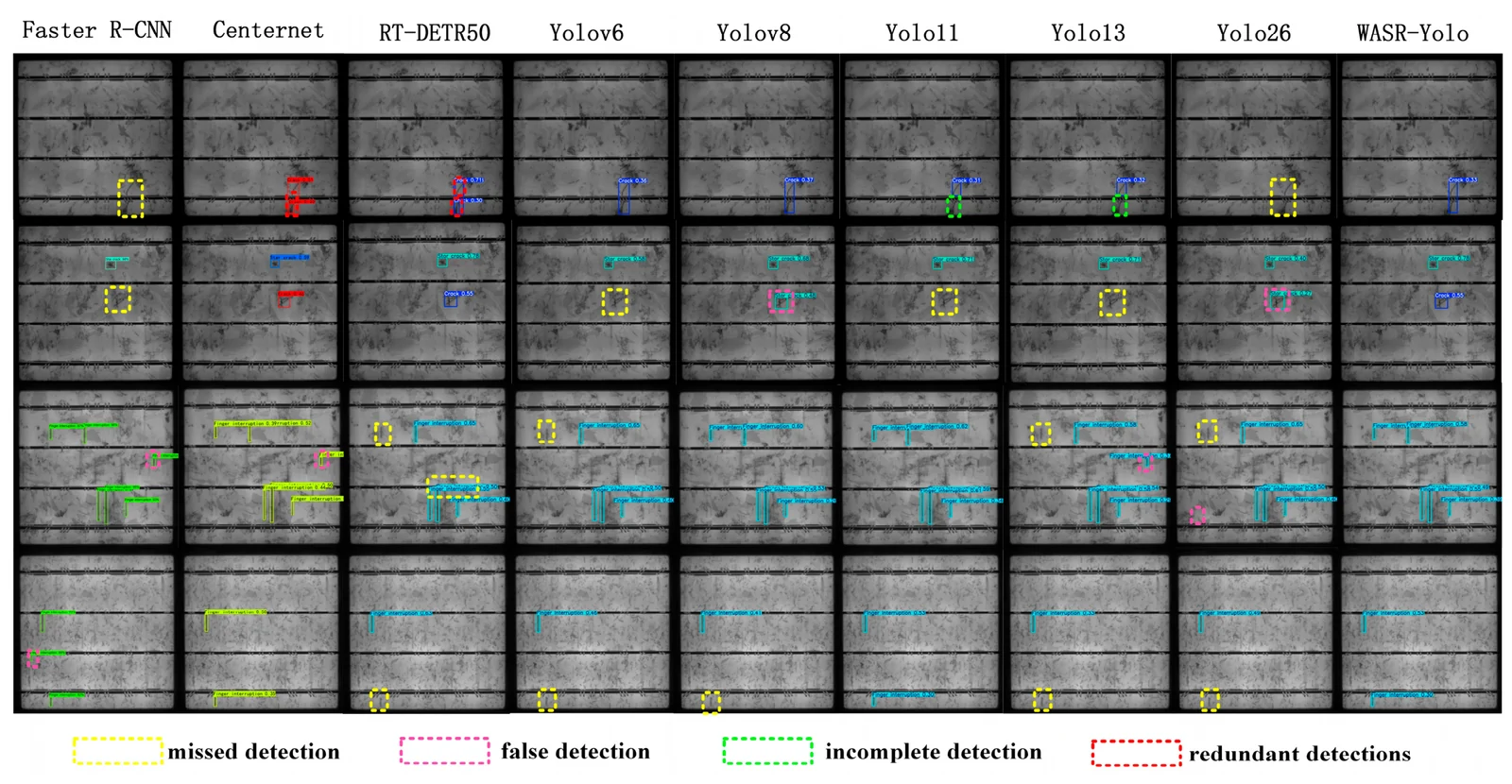
Visual comparison of small-defect detection results.

Figure 14 compares representative small-object detections from WASR and the baseline methods. Missed, false-positive, incomplete, and redundant detections are marked in yellow, pink, green, and red, respectively. The baseline methods produce false negatives and false positives when defects are small, weakly textured, blurred, or low in contrast. WASR recovers more complete defect regions, localizes their boundaries more precisely, and reduces missed detections. The visual comparison is consistent with the quantitative improvement in small-object precision.

### 4.7. Performance Under Complex Backgrounds and Low-Contrast Conditions

**Figure 15:**
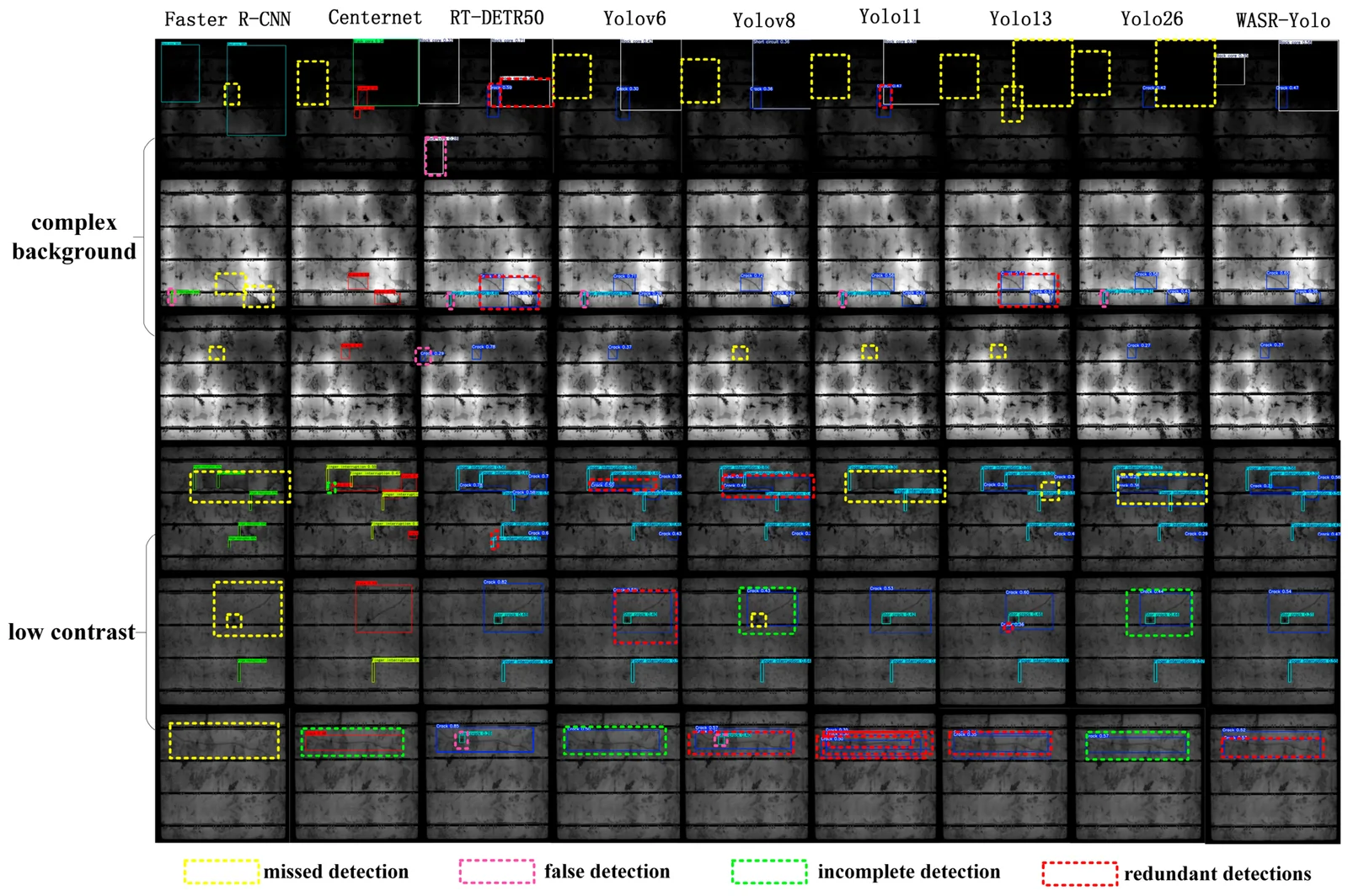
Detection results under complex backgrounds and low-contrast conditions.

Figure 15 compares representative detections under complex backgrounds and low contrast. In complex backgrounds, dense texture and nontarget structures obscure defect cues. RT-DETR50 and YOLOv6 produce false positives by assigning genuine defects to incorrect categories. Several other detectors, including Faster R-CNN, YOLOv6, and YOLOv26, also miss some defects. RT-DETR50 and YOLO13 also predict the same region repeatedly, reducing result reliability. By contrast, WASR suppresses background interference, retains discriminative responses in textured regions, and reduces false or repeated boxes while maintaining accurate localization.

In low-contrast scenes, weak grayscale differences make stable class boundaries difficult to learn, leading to incomplete or missed detections. In Figure 15, YOLOv6, YOLOv8, and YOLO11 produce redundant boxes, whereas Faster R-CNN and YOLO11 miss some targets. YOLOv8 and YOLOv26 identify only parts of some defect regions. WASR produces more continuous responses, covers defect regions more completely, and localizes weak boundaries more accurately.

Across both challenging cases, WASR provides more stable detection than the comparison methods. It reduces false and missed detections and improves coverage of defect regions in complex photovoltaic EL images. Together, these results demonstrate improved robustness and stability under both challenging conditions.

### 4.8. Practical Applicability

#### 4.8.1. Model Complexity and Modular Resource Consumption Analysis

The memory footprint and computational overhead of the proposed modules were evaluated using parameters, FLOPs, and model size. As shown in Table 10, although WADE and SHSA introduce additional wavelet and attention operations, the resulting increase in computational complexity remains limited, with only 2.62 M/6.4 G and 2.66 M/6.5 G parameters/FLOPs, respectively. Meanwhile, the CCFM neck reduces redundant feature fusion operations, enabling WASR to maintain a compact structure with only 1.96 M parameters, 5.6 G FLOPs, and a 4.13 MB model size. These results demonstrate that the proposed modules introduce limited memory and computational overhead, reducing their potential impact on inference latency and deployment on resource-constrained devices. At a batch size of 1, WASR achieved 58.98 FPS with a peak GPU memory usage of 197.53 MB, further verifying its efficient inference capability. Therefore, WASR achieves a favorable balance between detection accuracy and resource consumption, meeting the requirements of practical deployment.

#### 4.8.2. Embedded-Platform Deployment

**Figure 16:**
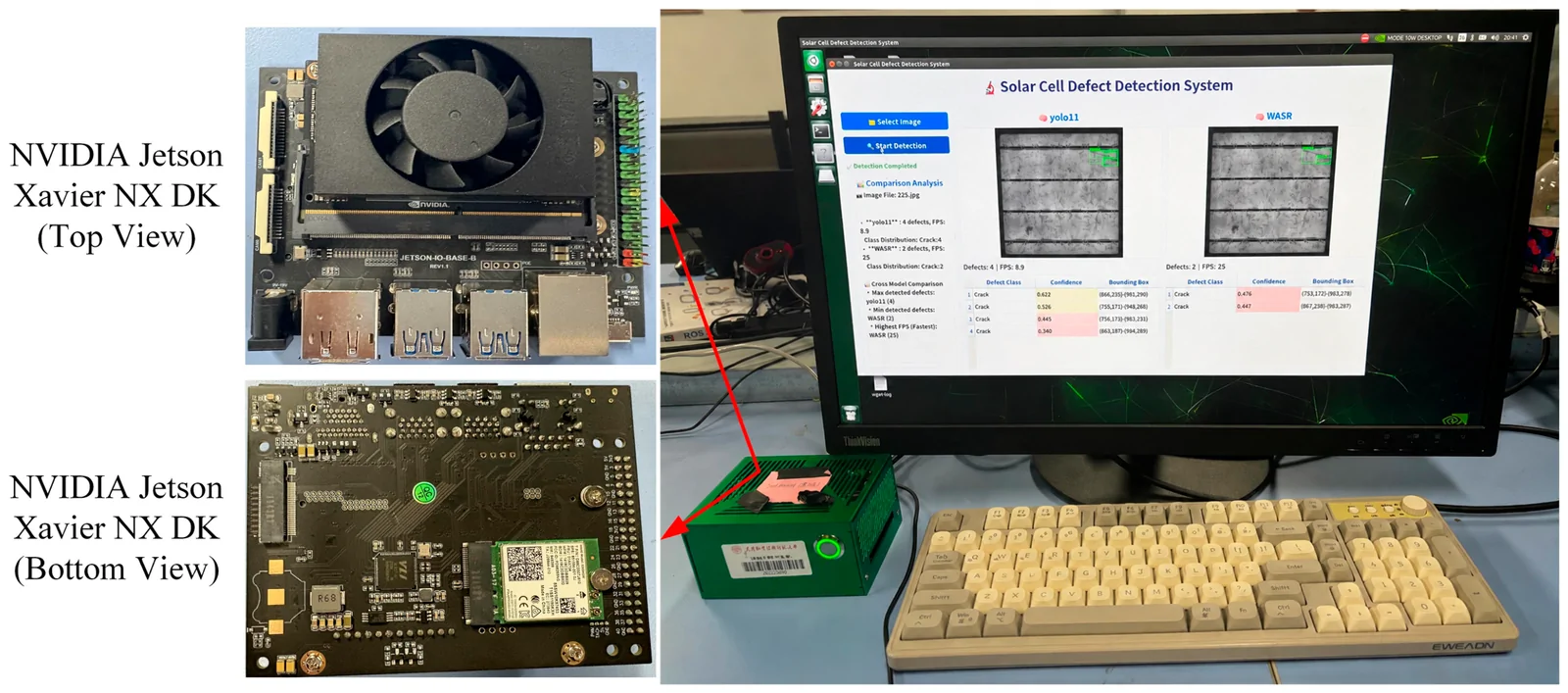
Embedded deployment platform.

The best-performing WASR model was deployed on an NVIDIA Jetson Xavier NX (NVIDIA Corporation, Santa Clara, CA, USA) to assess edge-platform performance. The PyTorch weights (.pt) were first exported to ONNX and then converted to an optimized TensorRT engine (.engine) for the Xavier NX. TensorRT graph optimization, layer fusion, and half-precision inference reduced embedded inference overhead. The deployment platform is shown in Figure 16.

**Figure 17:**
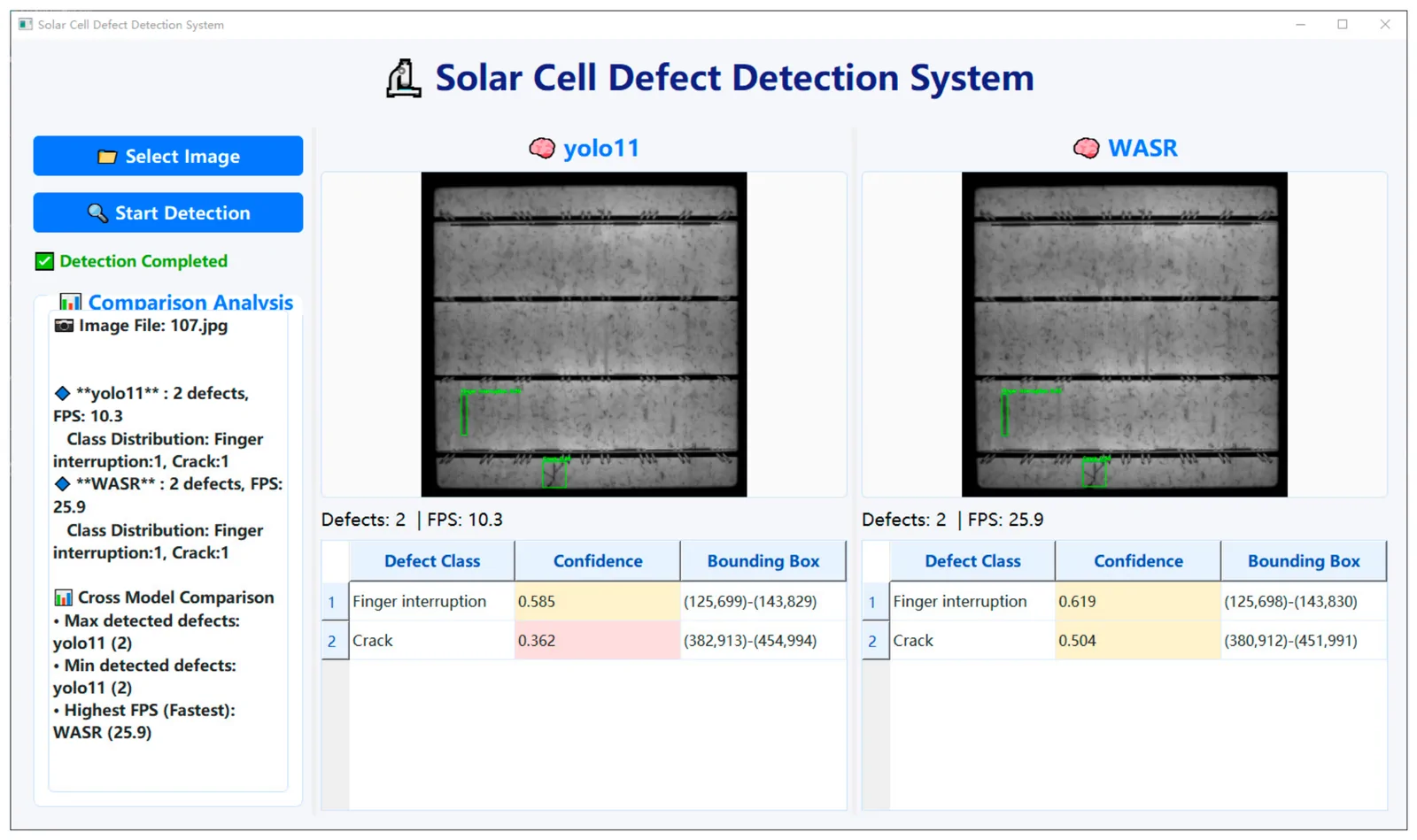
Host computer interface.

A host computer interface was also developed to display detection results and compare model performance. Under identical test conditions, both YOLO11 and WASR detected the sample defects (Figure 17). For the illustrated sample, inference speed increased from 10.3 FPS with YOLO11 to 25.9 FPS with WASR. The deployment test therefore shows that the lightweight design increases speed without losing defect-recognition capability.

## 5. Conclusions

A Wavelet-Aware Selective Reconstruction Network (WASR) was developed for photovoltaic EL defect detection under challenging conditions involving small defects, weak grayscale variations, and complex backgrounds. The architecture combines fine-detail preservation, background suppression, and lightweight detection. WADE preserves fine-grained small-defect features during downsampling. SHSA captures global contextual dependencies to distinguish defects from complex background texture. CCFM and SCDown improve multilevel interaction while reducing redundant computation. SRD-Head selectively reconstructs features to improve discrimination and localization stability. Rather than simply combining existing modules, the framework coordinates these components around the joint requirements of fine-grained feature preservation, complex-background suppression, and lightweight deployment.

WASR achieved 92.9% mAP@0.5 with 1.96 M parameters and 172 FPS, outperforming the YOLO11 baseline and the evaluated comparison models in terms of the overall accuracy–efficiency balance. Ablation, cross-dataset, repeated-seed, visualization, and deployment results support its effectiveness and practical potential. The quantitative and visual evidence also supports the framework’s robustness for practical photovoltaic EL defect detection. Future work will examine incremental learning and shape-aware localization to accommodate new defect types, evolving sample distributions, and elongated cracks.

## Figures and Tables

**Table 1:** Training hyperparameters.

Hyperparameter	Value
Learning Rate	0.01
Image Size	640 × 640
Momentum	0.9
Optimizer Type	SGD
Weight Decay	0.001
Batch Size	32
Epochs	200
Workers	8
Close_mosaic	0

**Table 2:** Quantitative comparison of feature quality under different downsampling methods.

Method	Entropy	Contrast	Edge Strength	Edge Ratio
Conventional downsampling	4.8787	32.9564	62.3679	0.0878
WADE	5.7710	40.4379	111.5500	0.3010

**Table 3:** Performance comparison of different detection algorithms.

Model	P/%	R/%	mAP@0.5/%	mAP@0.5:0.95/%	Params/M	FLOPs/G	FPS
Faster R-CNN	78.6	65.8	72.2	58.2	41.5	180	31
SSD	79.6	83.1	85.4	62.1	26.3	35	74
CenterNet	88.0	77.3	90.2	**71.9**	34.7	71	52
RT-DETR	80.8	83.3	86.7	65.4	41.9	136	43
YOLOv5	82.6	88.9	88.5	67.8	2.50	7.1	135
YOLOv6	83.1	86.3	87.8	69.3	4.23	11.8	102
YOLOv8	79.2	**89.5**	90.1	70.5	3.00	8.1	122
YOLOv10	87.6	84.9	90.3	70.5	2.26	6.5	158
YOLO11	80.4	82.6	90.5	71.3	2.58	6.3	149
YOLO13	81.7	88.5	90	70.3	2.44	6.1	162
YOLO26	82.9	78.3	83.9	67.1	2.61	5.7	165
WASR	**89.8**	88.6	**92.9**	71	**1.96**	**5.6**	**172**

Note: Bold values indicate the best results among all compared methods for each metric.

**Table 4:** Comparison of existing feature fusion methods.

Fusion Method	P/%	R/%	mAP@0.5/%	Params/M	FLOPs/G	FPS
PAN-FPN	80.4	82.6	90.5	2.58	6.3	149
BiFPN	81.2	81.3	88.3	2.84	7.4	128
Frefusion	83.4	89.2	91.3	2.43	6.6	162
CCFM	81.3	83.6	90.9	1.88	5.3	175

**Table 5:** Ablation results for different module combinations.

Experimental Group	WADE	SHSA	CCFM-SCDown	SRD-Head	mAP@0.5/%	mAP@0.5:0.95/%	Params/M
Control group					90.5	71.3	2.58
1	√				92.8	70.8	2.62
2		√			91.6	69.2	2.66
3			√		90.9	71.4	**1.88**
4				√	91.9	70.9	2.58
5	√	√			91.0	69.8	2.18
6	√	√	√		92.2	68.5	1.93
7		√	√	√	90.6	67.3	1.92
8	√		√	√	92.4	71	1.87
9	√	√		√	90.2	71.1	2.95
10	√	√	√	√	**92.9**	**71.6**	1.96

Note: The checkmark (√) indicates that the corresponding module is included in the method. Bold values indicate the best results among all compared methods for each metric.

**Table 6:** Detection performance on the PV-Multi-Defect dataset.

Model	P/%	R/%	mAP@0.5/%	mAP@0.5:0.95/%
Faster R-CNN	71.3	66.2	68.3	41.5
RT-DETR	71.6	70.6	72.5	49.8
YOLOv5	72.3	69.1	71.3	48.3
YOLOv6	73.6	70.8	70.7	47.8
YOLOv10	71.7	71.4	72.8	48.8
YOLO11	79.3	73.4	80.9	54.7
YOLO26	75.9	64.0	72.2	43.3
WASR	**82.6**	**75.0**	**83.4**	**56.9**

Note: Bold values indicate the best results among all compared methods for each metric.

**Table 7:** Results from three training runs with different random seeds.

Model	P/%	R/%	mAP@0.5/%	mAP@0.5:0.95/%
YOLO11	85.3 ± 4.2	84.5 ± 2.6	88.1 ± 1.1	68.73 ± 0.84
WASR	89.2 ± 2.8	86.5 ± 2.2	91.6 ± 0.3	69.6 ± 0.7

**Table 8:** Detection precision at different object scales using fixed-threshold partitioning.

Model	Small (P%)	Medium (P%)	Large (P%)
YOLO11	78.3%	39.5%	96.0%
WASR	84.9%	59.2%	97.3%

**Table 9:** Detection precision at different object scales using percentile partitioning.

Model	Small (P%)	Medium (P%)	Large (P%)
YOLO11	83.4%	69.8%	98.2%
WASR	86.7%	81.7%	99.0%

**Table 10:** Complexity metrics of models with different module combinations.

Model	Params/M	FLOPs/G	Model Size (MB)
YOLO11 + WADE	2.62	6.4	5.35
YOLO11 + SHSA	2.66	6.5	5.37
YOLO11 + CCFM-SCDown	1.88	5.3	3.98
YOLO11 + SRD-Head	2.58	6.3	5.23
WASR	1.96	5.6	4.13

## Data Availability

Data are contained within the article.

## Abbreviations

The following abbreviations are used in this manuscript:

EL	Electroluminescence
WASR	Wavelet-Aware Selective Reconstruction Network
WADE	Wavelet-Aware Downsampling Encoder
CCFM	Cross-Scale Context Fusion Module
SRD-Head	Selective Reconstruction Detection Head
mAP@0.5	Mean average precision at an IoU threshold of 0.5
FPS	Frames per second
PV	Photovoltaic
SVM	Support Vector Machine
R-CNN	Region-based Convolutional Neural Network
YOLO	You Only Look Once
SSD	Single Shot MultiBox Detector
CBAM	Convolutional Block Attention Module
GAM	Global Attention Mechanism
PVEL-AD	Photovoltaic Electroluminescence Vision Algorithm Defects
Params	Parameters
IoU	Intersection over union
SCDown	Spatial-Channel Downsampling
SHSA	Single-Head Self-Attention
DWT	Discrete Wavelet Transform
CNN	Convolutional Neural Network
SHViT	Single-Head Vision Transformer
Q	Query
K	Key
V	Value
H	Height
W	Width
C	Channel
PAN	Path Aggregation Network
ScConv	Spatial and Channel Reconstruction Convolution
SRU	Spatial Reconstruction Unit
CRU	Channel Reconstruction Unit
DWConv	Depthwise Convolution
CPU	Central Processing Unit
GPU	Graphics Processing Unit
VRAM	Video Random Access Memory
IDE	Integrated Development Environment
SGD	Stochastic Gradient Descent
P	Precision
R	Recall
AP	Average Precision
P–R	Precision–recall
FLOPs	Floating Point Operations
TP	True Positive
FP	False Positive
FN	False Negative
CUDA	Compute Unified Device Architecture
RTX	Ray Tracing Texel eXtreme
